# Identification of BXDC2 as a Key Downstream Effector of the Androgen Receptor in Modulating Cisplatin Sensitivity in Bladder Cancer

**DOI:** 10.3390/cancers13050975

**Published:** 2021-02-26

**Authors:** Guiyang Jiang, Yuki Teramoto, Takuro Goto, Taichi Mizushima, Satoshi Inoue, Hiroki Ide, Yujiro Nagata, Eiji Kashiwagi, Alexander S. Baras, George J. Netto, Zhiming Yang, Hiroshi Miyamoto

**Affiliations:** 1 Department of Pathology & Laboratory Medicine, University of Rochester Medical Center, Rochester, NY 14642, USA; jianggy@hotmail.com (G.J.); yuki_teramoto@urmc.rochester.edu (Y.T.); takuro_goto@urmc.rochester.edu (T.G.); mizu123shima@gmail.com (T.M.); inosts411@gmail.com (S.I.); y.nagata01@outlook.jp (Y.N.); yangzm2000@hotmail.com (Z.Y.); 2 James P. Wilmot Cancer Institute, University of Rochester Medical Center, Rochester, NY 14642, USA; 3 Department of Pathology, China Medical University, Shenyang 110122, Liaoning Province, China; 4 Department of Pathology, Johns Hopkins University School of Medicine, Baltimore, MD 21287, USA; h-ide@fc4.so-net.ne.jp (H.I.); eiji.pma@gmail.com (E.K.); baras@jhmi.edu (A.S.B.); gnetto@uabmc.edu (G.J.N.); 5 James Buchanan Brady Urological Institute, Johns Hopkins University School of Medicine, Baltimore, MD 21287, USA; 6 Department of Pathology, University of Alabama at Birmingham, Birmingham, AL 35249, USA; 7 Department of Urology, University of Rochester Medical Center, Rochester, NY 14642, USA

**Keywords:** androgen receptor, BXDC2, chemoresistance, cisplatin, immunohistochemistry, urothelial cancer

## Abstract

**Simple Summary:**

It remains unclear why chemotherapy is often ineffective in patients with bladder cancer. Meanwhile, we previously reported that male sex hormones (i.e., androgens) could considerably reduce the efficacy of cisplatin, an anti-cancer drug used as the first-line treatment against advanced bladder cancer. The present study aimed to investigate how androgen receptor signaling, which is activated by binding of androgenic hormones, modulates sensitivity to cisplatin treatment in bladder cancer, using cell line models and surgical specimens. We found that the expression levels of the androgen receptor and a molecule (BXDC2) were inversely correlated and that loss of BXDC2 was associated with cisplatin resistance. We thus provide evidence to suggest an underlying molecular mechanism responsible for androgen receptor-induced chemoresistance in bladder cancer.

**Abstract:**

Underlying mechanisms for resistance to cisplatin-based chemotherapy in bladder cancer patients are largely unknown, although androgen receptor (AR) activity, as well as extracellular signal-regulated kinase (ERK) signaling, has been indicated to correlate with chemosensitivity. We also previously showed ERK activation by androgen treatment in AR-positive bladder cancer cells. Because our DNA microarray analysis in control vs. AR-knockdown bladder cancer lines identified BXDC2 as a potential downstream target of AR, we herein assessed its functional role in cisplatin sensitivity, using bladder cancer lines and surgical specimens. BXDC2 protein expression was considerably downregulated in AR-positive or cisplatin-resistant cells. BXDC2-knockdown sublines were significantly more resistant to cisplatin, compared with respective controls. Without cisplatin treatment, BXDC2-knockdown resulted in significant increases/decreases in cell proliferation/apoptosis, respectively. An ERK activator was also found to reduce BXDC2 expression. Immunohistochemistry showed downregulation of BXDC2 expression in tumor (vs. non-neoplastic urothelium), higher grade/stage tumor (vs. lower grade/stage), and AR-positive tumor (vs. AR-negative). Patients with BXDC2-positive/AR-negative muscle-invasive bladder cancer had a significantly lower risk of disease-specific mortality, compared to those with a BXDC2-negative/AR-positive tumor. Additionally, in those undergoing cisplatin-based chemotherapy, BXDC2 positivity alone (*p* = 0.083) or together with AR negativity (*p* = 0.047) was associated with favorable response. We identified BXDC2 as a key molecule in enhancing cisplatin sensitivity. AR-ERK activation may thus be associated with chemoresistance via downregulating BXDC2 expression in bladder cancer.

## 1. Introduction

Urinary bladder cancer, mostly urothelial carcinoma, has been one of the most commonly diagnosed malignancies, especially in men [1,2]. In addition, the numbers of new bladder cancer cases and cancer deaths throughout the world have even risen from 429,800 and 165,100 estimated in 2010 [1] to 549,393 and 199,922 reported in 2018 [2], respectively. In particular, muscle-invasive bladder cancer is often associated with metastatic disease where the overall 5-year survival rate remains low (e.g., 5.5% [3]). Urothelial carcinoma also occurs in the upper urinary tract and is often (e.g., 60% [4]) invasive at the time of initial diagnosis.

While several immune checkpoint inhibitors have recently been approved for clinical use, cisplatin (CDDP)-based combination chemotherapy, such as “MVAC” (methotrexate (MTX)/vinblastine (VBL)/doxorubicin (DOX; Adriamycin)/CDDP), “GC” (gemcitabine (GEM)/CDDP), and “CMV” (CDDP/MTX/VBL), remains the mainstay of the treatment of locally advanced or metastatic urothelial carcinoma in a neoadjuvant or adjuvant setting [5,6]. Nonetheless, a considerable number of patients fail to respond to such systemic chemotherapy. Accordingly, the development of strategies for overcoming chemoresistance constitutes a goal with critical clinical implications.

The mechanisms for CDDP resistance remain poorly understood, although multiple molecular pathways, including extracellular signal-regulated kinases (ERKs) and PTEN, have been suggested to involve the modulation of the cytotoxic and anti-proliferative potential of CDDP [7,8]. We have additionally demonstrated that activation of androgen receptor (AR) is associated with resistance to CDDP therapy in bladder cancer [9], while an increasing amount of evidence indicates an important role of AR in promoting both of two distinct steps/events, urothelial tumorigenesis and tumor progression [10]. In one of our studies, epidermal growth factor receptor (EGFR)-ERK signaling activation by AR in bladder cancer cells has also been documented [11]. Interestingly, our recent observations have suggested that AR activity in bladder cancer cells correlates with sensitivity to other conventional non-surgical treatments, such as intravesical BCG immunotherapy [12] and radiotherapy [13].

Our recent DNA microarray analysis identified candidate genes, including *BXDC2* whose expression was significantly upregulated in an AR-knockdown bladder cancer subline, compared with AR-positive control cells [12]. To further elucidate how AR signals modulate chemosensitivity in urothelial cancer, we here investigated the functional role of a potential downstream effector, BXDC2, known to involve ribosome biogenesis, using bladder cancer cell lines and surgical specimens.

## 2. Results

### 2.1. Associations between AR and BXDC2 Expression

We had recently employed DNA microarray analysis in control AR-positive UMUC3 versus a UMUC3 subline stably expressing AR-short hairpin RNA (shRNA) [12]. Of those expressed at absolutely high levels, several candidate genes were examined if their expression was not only upregulated in AR-knockdown cells but also downregulated in CDDP-resistant cells. Indeed, a quantitative PCR confirmed the significant increase or decrease in the levels of BXDC2 expression in AR-knockdown or CDDP-resistant subline, respectively, compared with control cells. We thus decided to further investigate the functions of BXDC2, whose roles in neoplastic diseases were largely unknown.

**Figure 1:**
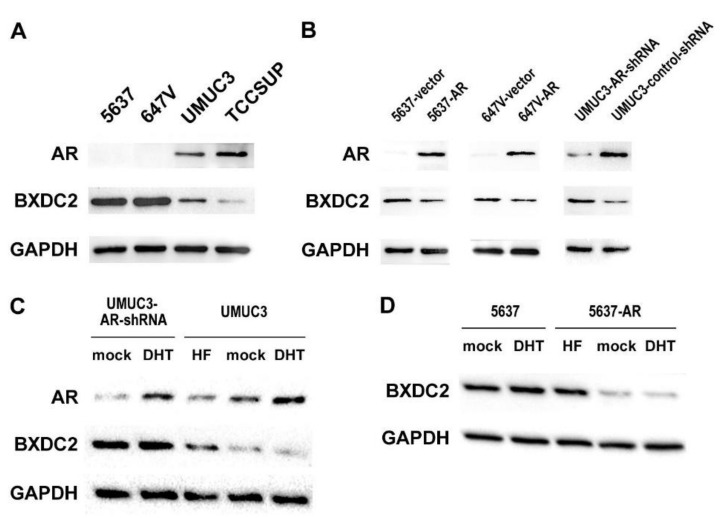
Relationship between androgen receptor (AR) and BXDC2 expression in bladder cancer cells. (**A**) Western blotting of AR and BXDC2 in 5637, 647V, UMUC3, and TCCSUP. (**B**) Western blotting of AR and BXDC2 in 5637-vector vs. 5637-AR, 647V-vector vs. 647V-AR, and UMUC3-AR-shRNA vs. UMUC3-control-shRNA. (**C**) Western blotting of AR and BXDC2 in UMUC3-AR-shRNA or UMUC3 cultured for 24 h with ethanol (mock), 10 nM DHT, or 5 µM HF. (**D**) Western blotting of BXDC2 in 5637 or 5637-AR cultured for 24 h with ethanol (mock), 10 nM DHT, or 5 µM HF. GAPDH served as a loading control.

We first examined the expression of BXDC2 in 4 human bladder cancer lines that were known to be AR-negative (i.e., 5637, 647V) or AR-positive (i.e., UMUC3, TCCSUP) [14]. Western blot detected BXDC2 signals in all the cell lines, and the levels were higher in AR-negative cells than in AR-positive cells (Figure 1A). We then compared the BXDC2 levels in AR-negative/knockdown versus AR-positive/overexpression sublines. Consistent with the DNA microarray data [12], BXDC2 expression was upregulated in no or low AR cells (Figure 1B). We further assessed the effects of androgen (i.e., dihydrotestosterone (DHT)) and anti-androgen (i.e., hydroxyflutamide (HF)) on BXDC2 expression. DHT treatment (vs. mock treatment) did not reduce the level of BXDC2 expression in UMUC3-AR-shRNA cells, whereas HF treatment (vs. mock treatment) considerably induced it in UMUC3 cells (Figure 1C). Similarly, no significant effect of DHT and a considerable stimulatory effect of HF on BXDC2 expression were seen in 5637 and 5637-AR, respectively (Figure 1D). Thus, the expression of BXDC2 was inversely correlated with the expression/activity of AR in bladder cancer cells.

### 2.2. Associations between BXDC2 Expression and CDDP Sensitivity

**Figure 2:**
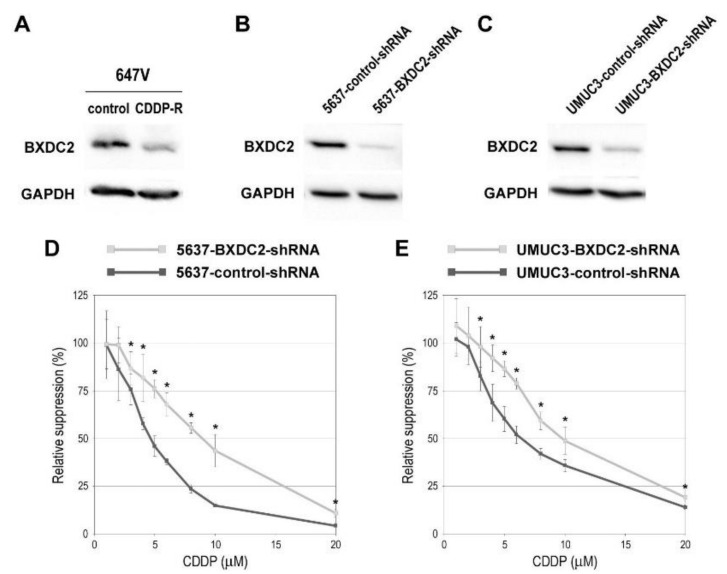
Effects of BXDC2 knockdown on cisplatin (CDDP) cytotoxicity in bladder cancer cells. Western blotting of BXDC2 in 647V-control vs. 647V-CDDP-resistant (R) sublines (**A**), 5637-control-shRNA vs. 5637-BXDC2-shRNA sublines (**B**), or UMUC3-control-shRNA vs. UMUC3-BXDC2-shRNA sublines (**C**). GAPDH served as a loading control. MTT assay in 5637-control-shRNA vs. 5637-BXDC2-shRNA sublines (**D**) or UMUC3-control-shRNA vs. UMUC3-BXDC2-shRNA sublines (**E**) cultured for 72 h in the presence of various concentrations (0–20 µM) of CDDP. Cell viability is presented relative to that of each subline without CDDP treatment. Each value represents the mean (± SD) from a total of 6 determinants. * *p* < 0.05 (vs. control-shRNA).

We next assessed the impact of BXDC2 expression on sensitivity to CDDP treatment in bladder cancer cells. Western blotting confirmed significant downregulation of BXDC2 expression in CDDP-resistant cells, compared with control cells (Figure 2A). To compare the cytotoxic effects of CDDP in BXDC2-positive versus BXDC2-negative cells, we silenced BXDC2. As expected, the levels of BXDC2 were substantially lower in 5637 (Figure 2B) and UMUC3 (Figure 2C) sublines stably expressing BXDC2-shRNA than in respective control-shRNA-expressing sublines. The methyl-thiazolyl-disphenyl-tetrazolium bromide (MTT) assay then showed that 5637-BXDC2-shRNA (Figure 2D) and UMUC3-BXDC2-shRNA (Figure 2E) were significantly more resistant to CDDP (e.g., 3–20 µM), compared with respective control sublines. The IC50s were: 4.7 µM (in 5637-control-shRNA) vs. 8.6 µM (in 5637-BXDC2-shRNA); and 6.7 µM (in UMUC3-control-shRNA) vs. 9.9 µM (in UMUC3-BXDC2-shRNA). In these assays, induction of cell growth by BXDC2-knockdown (see Figure 4A), irrespective of CDDP, was excluded by comparing with the viability of each mock-treated subline (with no CDDP treatment).

**Figure 3:**
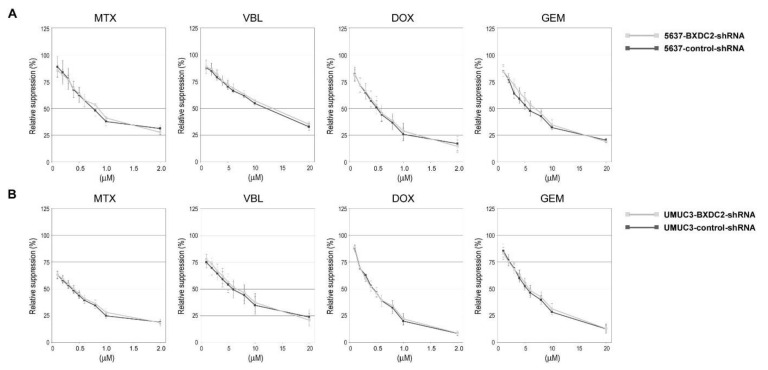
Effects of BXDC knockdown on the cytotoxicity of various anti-cancer agents in bladder cancer cells. MTT assay in 5637-control-shRNA vs. 5637-BXDC2-shRNA sublines (**A**) or UMUC3-control-shRNA vs. UMUC3-BXDC2-shRNA sublines (**B**) cultured for 72 h in the presence of MTX (0–2.0 µM), VBL (0–20 µM), DOX (0–2.0 µM), or GEM (0–20 µM). Cell viability is presented relative to that of each subline without anti-cancer drug treatment. Each value represents the mean (± SD) from at least 3 determinants.

To see if BXDC2 could modulate the cytotoxic effects of other chemotherapeutic drugs used for the treatment of bladder cancer, the cell viability in the presence of various concentrations of MTX, VBL, DOX, or GEN was compared in the control-shRNA vs. BXDC2-shRNA sublines. However, there were no significant differences in sensitivity to these anti-cancer agents between the control and knockdown sublines derived from 5637 (Figure 3A) or UMUC3 (Figure 3B).

### 2.3. Role of BXDC2 in Cell Growth

**Figure 4:**
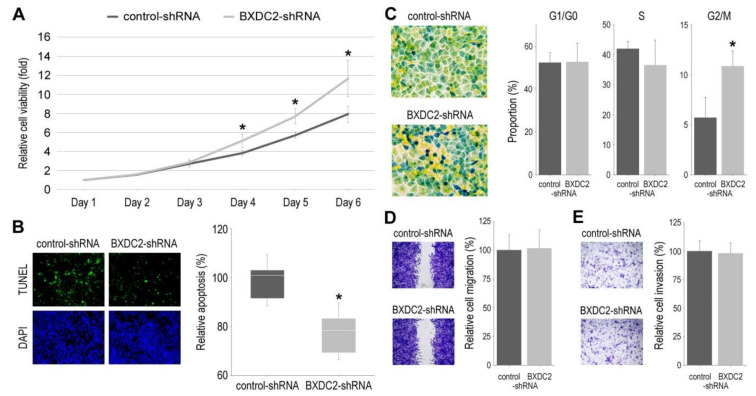
Effects of BXDC2 knockdown on the growth of bladder cancer cells. (**A**) MTT assay in 5637-control-shRNA vs. 5637-BXDC2-shRNA sublines cultured for 1–6 days. Cell viability is presented relative to that of control subline at day 1. Each value represents the mean (± SD) from a total of 6 determinants. (**B**) TUNEL assay in 5637-control-shRNA vs. 5637-BXDC2-shRNA sublines. Apoptosis counted as a percentage of at least 500 cells is presented relative to that of control subline. Each value represents the median (± SE) from a total of 16 determinants. (**C**) Cell cycle phase analysis in 5637-control-shRNA vs. 5637-BXDC2-shRNA sublines. Color changes are associated with cells in G0/G1 (yellow), S (green), and G2/M (blue) phases. Proportion of each phase counted as a percentage of more than 1.4 × 10^6^ cells represents the mean (+ SD). (**D**) Wound-healing assay in 5637-control-shRNA vs. 5637-BXDC2-shRNA sublines gently scratched and cultured for 24 h. Cell migration determined by the rate of cells filling the wound area is presented relative to that of control subline. Each value represents the mean (+ SD) from a total of 16 determinants. (**E**) Transwell invasion assay in 5637-control-shRNA vs. 5637-BXDC2-shRNA sublines. Cell invasion determined by counting the number of invaded cells in the lower chamber under a microscope is presented relative to that of control subline. Each value represents the mean (+ SD) from a total of 10 determinants. * *p* < 0.05 (vs. control-shRNA).

Using control-shRNA vs. BXDC2-shRNA sublines, we assessed the role of BXDC2 in the cell proliferation (via MTT assay (Figure 4A)), apoptosis (via TUNEL assay (Figure 4B)), cell cycle (Figure 4C), cell migration (via wound-healing assay (Figure 4D)), and cell invasion (via transwell invasion assay (Figure 4E)). In these assays, BXDC2-knockdown resulted in significant increases in cell viability and G2/M population, as well as a significant decrease in apoptosis. However, there were no significant differences in the G1/G0 and S phases or the ability of cell migration or invasion between the two sublines.

### 2.4. Involvement of BXDC2 in AR-ERK Signaling

**Figure 5:**
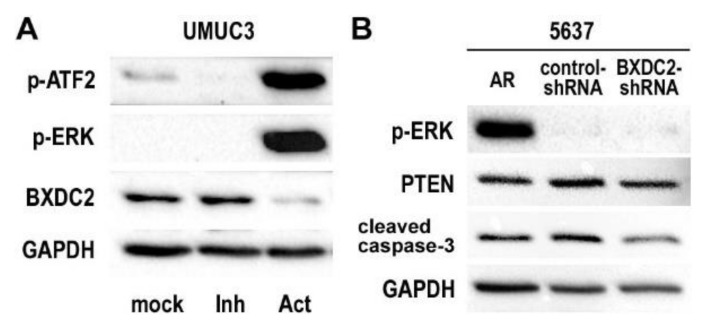
Expression of potential BXDC2 upstream and downstream proteins in bladder cancer cells. (**A**) Western blotting of p-ATF2, p-ERK, and BXDC2 in UMUC3 cultured with ethanol (mock), SCH 772984 (“Inh”; 4 nM for 6 h), or C6 ceramide (“Act”; 10 µM for 24 h). (**B**) Western blotting of p-ERK, PTEN, and cleaved caspase-3 in 5637-AR, 5637-control-shRNA, and 5637-BXDC2-shRNA sublines. GAPDH served as a loading control.

As aforementioned, AR activation has been implicated in CDDP resistance in bladder cancer cells [9]. Androgens have also been shown to activate ERK [11], as well as ATF2 [15] which can be phosphorylated in response to phospho-ERK signals, in bladder cancer cells via the AR pathway. To further determine if BXDC2 is involved in the ERK signaling, Western blotting was performed. In AR-positive UMUC3 cells, an ERK activator dramatically reduced the expression of BXDC2 (while inducing that of p-ATF2 and p-ERK), and an ERK inhibitor slightly induced BXDC2 expression (Figure 5A), suggesting that BXDC2 could represent a downstream target of ERK. However, there were no significant differences in the expression of p-ERK (and PTEN) between control-shRNA and BXDC2-shRNA cells, suggesting that BXDC2 is not an upstream of ERK (or PTEN), while BXDC2-knockdown resulted in a decrease in the expression of cleaved caspase-3 (Figure 5B).

### 2.5. Expression of BXDC2 in Bladder Cancer Specimens

**Figure 6:**
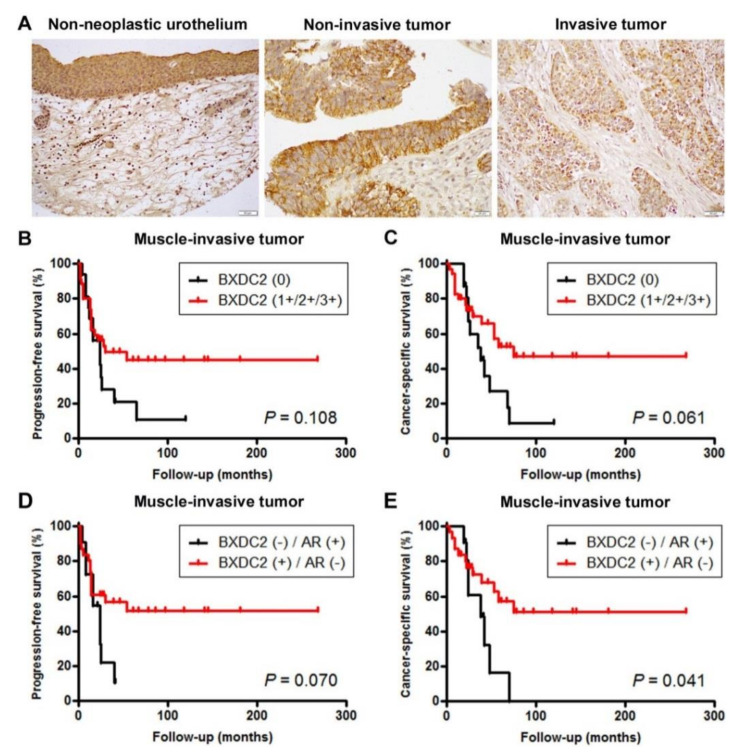
Immunohistochemistry of BXDC2 in surgical specimens. (**A**) Expression of BXDC2 in non-neoplastic urothelium (original magnification: ×200), non-invasive urothelial tumor (original magnification: ×400), and invasive urothelial tumor (original magnification: ×200). Immunoreactivity is seen mainly in the cytoplasm of urothelial cells. Kaplan–Meier analysis for progression-free survival (**B**) or cancer-specific survival (**C**) in patients with BXDC2-negative (*n* = 16) vs. BXDC2-positive (*n* = 35) muscle-invasive tumor. Kaplan–Meier analysis for progression-free survival (**D**) or cancer-specific survival (**E**) in patients with BXDC2-negative/AR-positive (*n* = 9) vs. BXDC2-positive/AR-negative (*n* = 30) muscle-invasive tumor.

We stained immunohistochemically for BXDC2 in two separate sets of bladder tissue microarray (TMA). Positive signals were detected predominantly in the cytoplasm of non-neoplastic and neoplastic epithelial cells (Figure 6A; also see Supplementary Figure S1).

In the first set of TMA consisting of 129 cases of urothelial neoplasms and corresponding 72 non-neoplastic normal-appearing urothelial tissues, BXDC2 was positive in 65 (90%) of non-neoplastic and 99 (77%) of neoplastic specimens (Table 1). Thus, the rate of BXDC2 positivity was significantly lower in tumors than in benign tissues. In addition, BXDC2 expression was insignificantly or significantly downregulated in high-grade (71%), muscle-invasive (69%), and lymph node-positive (46%) tumors, compared with lower grade (86%), non-muscle-invasive (82%), and pN0 (81%) cases, respectively. Meanwhile, in our previous study [16], AR had been found to be immunoreactive in 58 (45%) of the present 129 tumors. There was thus an inverse correlation between BXDC2 and AR expression (*p* = 0.007) (see Supplementary Figure S1).

We then performed Kaplan–Meier analysis coupled with the log-rank test to assess possible associations of BXDC2 expression with patient outcomes. In patients with non-muscle-invasive disease, loss of BXDC2 expression tended to be associated with the risk of tumor recurrence (*p* = 0.074) or progression (*p* = 0.056) (Supplementary Figure S2). In patients with muscle-invasive disease, loss of BXDC2 expression tended to be associated with the risk of cancer-specific mortality (*p* = 0.061; Figure 6C), but not that of tumor progression (*p* = 0.106; Figure 6B). When considering the expression status of both BXDC2 and AR, there were no significant differences in recurrence-free survival (*p* = 0.299) or progression-free survival (*p* = 0.406) in patients with non-muscle-invasive disease between BXDC2-negative/AR-positive versus BXDC2-positive/AR-negative tumors. However, patients with BXDC2-negative/AR-positive muscle-invasive tumor had an insignificantly (*p* = 0.070) or significantly (*p* = 0.041) higher risk of disease progression (Figure 6D) or cancer-specific mortality (Figure 6E), respectively.

In another set of TMA consisting of muscle-invasive bladder cancer specimens from those who had subsequently undergone CDDP-based neoadjuvant chemotherapy, BXDC2 was positive in 26 (60%) of 43 cases. These BXDC2-positive cases included 13 (76%) responders and 13 (50%) non-responders to the neoadjuvant chemotherapy (Table 2). Thus, loss of BXDC2 expression tended to be associated with chemoresistance (*p* = 0.083). Additionally, when the status of AR expression stained in our previous study [9] was considered, the association of chemosensitivity with BXDC2/AR expression (i.e., BXDC2-negative/AR-positive vs. BXDC2-positive/AR-negative) was statistically significant (*p* = 0.047; Table 3).

## 3. Discussion

Although resistance to CDDP-based chemotherapy is commonly seen in patients with urothelial cancer, its underlying mechanisms remain to be established. Meanwhile, AR activation in bladder cancer cells has been suggested to induce chemoresistance [9,17,18]. In the present study, we further investigated the role of BXDC2, whose expression could be downregulated by AR, in CDDP resistance, using bladder cancer cell lines as well as surgical specimens.

BXDC2, also named BRIX1, belongs to the Imp4 superfamily and is known to play an important role in ribosome biogenesis via involving, for instance, RNA processing, subunit assembly, and export to the cytoplasm [19,20,21,22]. A recent study showed that BRIX1 expression could be regulated by transcription factors MYC1 and MYC2 [23]. A particulate matter, PM2.5, was also found to reduce the expression level of the *BRIX1* gene in myocardial cells [24]. Moreover, genome-wide association analysis data suggested the potential role of BRIX1 in disease resistance in chicken [25]. By contrast, very little is known about the functional role of BXDC2 in the development and progression of neoplastic diseases. Only a study has demonstrated elevated expression of *BXDC2* gene in immortalized lung fibroblasts, lung cancer cell lines, and non-small cell lung cancer tissues [26], suggesting its oncologic role. We here found significant induction in the proliferation of BXDC2-knockdown cells presumably via increasing G2/M phase and decreasing apoptosis, suggesting the preventive role of BXDC2 in the growth of bladder cancer cells. Immunohistochemistry in surgical specimens further showed decreased expression of BXDC2 in bladder tumors, compared with non-neoplastic urothelial tissues, especially in high-grade, muscle-invasive, and/or lymph node-positive cases. There was also a tendency to associate between loss of BXDC2 expression and a higher risk of disease recurrence (in non-muscle-invasive tumors) or disease-specific mortality (in muscle-invasive tumors). These findings may thus indicate that BXDC2 functions as a tumor suppressor in bladder cancer. However, in vitro assays demonstrated no significant effects of BXDC2 expression on the migration and invasion of bladder cancer cells.

Again, both AR and ERK signals have been associated with CDDP resistance [7,8,9]. Our previous study additionally indicated that ERK could be activated by AR in bladder cancer cells [11]. However, it was unclear how AR-ERK signaling induced chemoresistance. In the present study, we first showed that BXDC2 expression increased sensitivity to CDDP treatment in bladder cancer cells. Then, an ERK activator considerably reduced the levels of BXDC2 expression, while BXDC2-knockdown failed to affect the expression of p-ERK. Thus, CDDP resistance via the AR → ERK → BXDC2 signaling pathway in bladder cancer cells was strongly suggested. We additionally demonstrated that BXDC2 had no significant impact on modulating sensitivity to other chemotherapeutic drugs included in the standard regimens for the treatment of advanced bladder cancer, such as MTX, VBL, DOX, and GEM. Meanwhile, no studies have revealed significant sex-related differences in the prognosis of bladder cancer patients who undergo systemic chemotherapy. This may be due to a similar role of estrogen signaling in modulating chemosensitivity (e.g., induction of chemoresistance via the estrogen receptor-β pathway) [27]. Modulation of BXDC2 expression by estrogen signaling may thus need to be further investigated.

In addition to chemoresistance, AR signaling in urothelial cancer cells has been implicated in urothelial tumorigenesis and tumor progression [10], the former of which may clearly explain male dominance in the incidence of bladder cancer. We have specifically demonstrated that knockdown of AR or treatment with AR antagonists considerably inhibits both of them [14,28,29] and that AR signals either activate oncogenic molecules, such as ATF2 [15], β-catenin [30], EGFR/ERK/Akt [11], ELK1 [31], and NF-κB [32], in bladder cancer cells or inactivate tumor suppressors, such as GATA3 [33], FOXO1 [34], and UGT1A [35], in non-neoplastic urothelial cells. The expression of AR and BXDC2 in bladder cancer lines and tissue specimens was then found to be inversely correlated, suggesting that BXDC2 could be a downstream effector of AR. Although, as described above, AR does not appear to directly regulate the expression of BXDC2, it is of importance to further investigate its functional role especially in the development of urothelial cancer.

## 4. Materials and Methods

### 4.1. Cell Lines

Human urothelial carcinoma cell lines, 5637, 647V, UMUC3, and TCCSUP, were originally obtained from the American Type Culture Collection and recently authenticated by the institutional core facility. Sublines stably expressing human wild-type AR (or vector only) or AR-shRNA (or control-shRNA) were established in our previous studies (e.g., 5637-AR [11], 647V-AR [33], UMUC3-AR-shRNA [11]). Similarly, BXDC2-shRNA (MISSION^®^ shRNA Bacterial Glycerol Stock, TRCN0000122361, Millipore Sigma) was stably expressed in 5637 and UMUC3 lines. In addition, a 647V subline resistant to CDDP (or a control cultured for the same period without CDDP) was established by ≥12-week culture with low/increasing doses of CDDP in our previous study [9]. These parental lines and sublines were maintained in DMEM (ThermoFisher) supplemented with 10% fetal bovine serum (FBS) and then cultured in phenol red-free medium supplemented with 5% FBS at least 24 h before experimental treatment.

### 4.2. Antibodies and Chemicals

We obtained anti-AR (N-20), anti-BXDC2 (C-1), and anti-GAPDH (6c5) antibodies, and anti-p-ATF2 (Thr71), anti-p-p44/42 MAPK (ERK) (Thr202/Tyr204), anti-PTEN (D4.3), and anti-cleaved caspase-3 (Asp175) antibodies from Santa Cruz Biotechnology and Cell Signaling Technology, respectively. CDDP, DHT, and HF were from Sigma-Aldrich, while MTX, VBL, DOX, GEM, C6 ceramide, and SCH 772984 were from Cayman Chemical.

### 4.3. Western Blotting

Equal amounts of proteins (30 µg) obtained from cell extracts were subjected to electrophoresis with 10% sodium dodecyl sulfate-polyacrylamide gel, which was transferred to polyvinylidene difluoride membrane electronically. After blocking with 0.03–0.3% Blotting-Grade Blocker (BioRad), the membrane was incubated with a primary antibody at 4 °C overnight, followed by 1-h incubation with an HRP-conjugated secondary antibody (Cell Signaling Technology) at room temperature. Chemiluminescent signals were generated by Clarity western ECL substrate and detected by ChemiDOC™ MP (Bio-Rad).

### 4.4. Cell Proliferation

We used the MTT assay to assess cell viability. Cells (5 × 10^3^/well) seeded in 96-well tissue culture plates were cultured for up to 144 h and then incubated with 0.5 mg/mL of MTT (Sigma-Aldrich) for 3 h at 37 °C. MTT was dissolved by dimethyl sulfoxide, and the absorbance at 570 nm was measured.

### 4.5. Apoptosis and Cell Cycle Analysis

The TUNEL assay was conducted on cell-burdening coverslips, using the DeadEnd Fluorometric TUNEL system (Promega), followed by counterstaining for DNA with 4′,6-diamidino-2-phenylindole (DAPI). Apoptotic index was determined in the cells visualized by the fluorescence microscopy. For cell cycle phase quantification, the Cell Cycle Assay Cell-Clock™ (Biocolor) was used according to the manufacturer’s protocol. Data were analyzed, using ImageJ software (National Institutes of Health).

### 4.6. Cell Migration

Scratch wound-healing assay was adapted to evaluate the ability of cell migration. Cells at a density of ≥90% confluence in 12-well tissue culture plates were scratched manually with a sterile 200 μL plastic pipette tip. The wounded monolayers of the cells were allowed to heal in serum-free medium for 24 h, and the width of the wound area was monitored with an inverted microscope. The normalized cell-free area in photographed pictures (24 h/0 h) was quantitated, using the ImageJ.

### 4.7. Bladder TMA and Immunohistochemistry

Two sets of TMA consisting of retrieved bladder tissue specimens obtained by transurethral surgery performed at the Johns Hopkins Hospital were constructed previously [9,16]. None of the patients had received therapy with radiation or anti-cancer drugs prior to the collection of the tissues. The first set consisted of 129 cases of urothelial neoplasm along with paired normal-appearing bladder tissues from patients with tumor. All 51 patients with high-grade muscle-invasive tumor ultimately underwent radical cystectomy without neoadjuvant therapy. Tumor progression was evaluated separately in 78 cases with non-muscle-invasive tumor (i.e., development of high-grade (from low-grade), invasive (from non-invasive), and/or muscle-invasive tumors) and 51 cases with muscle-invasive tumor (i.e., development of recurrent or metastatic tumors after cystectomy). The second set consisted of 43 cases of high-grade muscle-invasive urothelial carcinoma that had subsequently received GC neoadjuvant chemotherapy (i.e., ≥3 cycles without dose reduction or ≥4 cycles with dose reduction) prior to radical cystectomy, including 17 responders and 26 non-responders, as defined previously [9,36].

Immunohistochemical staining was performed on the 5-µm sections, using a primary antibody to BXDC2, as we described previously [7,9,15]. All stains were manually quantified by a single pathologist (H.M.) who was blinded to sample identify. The final scores (range: 0–12) calculated by multiplying the percentage of immunoreactive cells (0% = 0; 1–10% = 1; 11–50% = 2; 51–80% = 3; 81–100% = 4) by staining intensity (negative = 0; weak = 1; moderate = 2; strong = 3) were considered negative (0; score < 2), weakly positive (1+; 2 ≤ score ≤ 4), moderately positive (2+; 4 < score ≤ 8), and strongly positive (3+; score > 8).

### 4.8. Statistical Analysis

Fisher’s exact test or the Chi-square test was used to evaluate the associations between categorized variables. Student’s *t*-test was used to compare numerical data. Survival rates in patients were calculated by the Kaplan–Meier method and comparison was made by log-rank test. *p* < 0.05 was considered to be statistically significant.

## 5. Conclusions

We identified BXDC2 as a key downstream effector of AR in modulating CDDP sensitivity in bladder cancer. Specifically, AR-ERK activation was suggested to induce chemoresistance via downregulating BXDC2 expression. While BXDC2 activators are not currently available, our data further support that concurrent anti-androgen therapy has the potential of being a means of chemosensitization, especially in male patients with AR-positive bladder tumor. In addition, because BXDC2-negative/AR-positive tumors were strongly associated with resistance to CDDP-based chemotherapy, the expression status of BXDC2, along with that of AR, might serve as a predictor of chemosensitivity in patients with bladder cancer. Meanwhile, further investigation of BXDC2 activity in animal models for bladder cancer is required to address the feasibility of future therapeutic intervention.

## Figures and Tables

**Table 1:** Correlations between BXDC2 expression in bladder cancers and their clinicopathological profile.

Clinicopathology	*n*	Expression Levels of BXDC2				*p* Value
		Negative	Positive			
		0	1+	2+	3+	0 vs. 1+/2+/3+
**Tissues**						0.022
Non-neoplastic urothelium	72	7 (10%)	33 (46%)	21 (29%)	11 (15%)	
Urothelial neoplasm	129	30 (23%)	61 (47%)	29 (22%)	9 (7%)	
**Tumor Grade**						0.056
PUNLMP + LG	50	7 (14%)	29 (58%)	11 (22%)	3 (6%)	
HG	79	23 (29%)	32 (41%)	18 (23%)	6 (8%)	
**Pathologic Stage**						0.091
Non-muscle-invasive	78	14 (18%)	39 (50%)	18 (23%)	7 (9%)	
Muscle-invasive	51	16 (31%)	22 (43%)	11 (22%)	2 (4%)	
**Lymph Node Involvement**						0.031
pN0	36	7 (19%)	17 (47%)	11 (31%)	1 (3%)	
pN1-3	13	7 (54%)	4 (31%)	1 (8%)	1 (8%)	
**AR expression**						0.007
Negative	71	10 (14%)	43 (61%)	13 (18%)	5 (7%)	
Positive	58	20 (34%)	18 (31%)	16 (28%)	4 (7%)	

PUNLMP: papillary urothelial neoplasm of low malignant potential; LG: low-grade urothelial carcinoma; HG: high-grade urothelial carcinoma; AR: androgen receptor.

**Table 2:** BXDC2 expression in bladder cancer and response to chemotherapy.

Chemoresponse	*n*	BXDC2 Expression		*p* Value
		0	1+/2+/3+	
Responders	17	4 (24%)	13 (76%)	0.083
Non-responders	26	13 (50%)	13 (50%)	

**Table 3:** The expression of BXDC2 and AR in bladder cancer and response to chemotherapy.

Chemoresponse	*n*	BXDC2(−)/AR(+)	BXDC2 (+)/AR (−)	*p* Value
Responders	13	2 (15%)	11 (85%)	0.047
Non-responders	18	9 (50%)	9 (50%)	

## Data Availability

The data presented in this study are available on request from the corresponding author but are not publicly available due to privacy and/or ethical restrictions.

## Supplementary Materials

The following are available online at https://www.mdpi.com/2072-6694/13/5/975/s1, Figure S1: The expression of BXDC2 and AR in bladder cancer tissues. Figure S2: Kaplan–Meier analysis for recurrence-free survival or progression-free survival in patients with BXDC2-negative (*n* = 14) vs. BXDC2-positive (*n* = 64) non-muscle-invasive tumor. Figure S3: The whole Western blot images for Figure 1, Figure 2 and Figure 5.
