# Supplementary material for: Identification of BXDC2 as a Key Downstream Effector of the Androgen Receptor in Modulating Cisplatin Sensitivity in Bladder Cancer
Source: Cancers (Basel). 2021 Feb 26;13(5):975. doi: 10.3390/cancers13050975 (PMC7956795; doi:10.3390/cancers13050975)
Supplement: Supplementary file 1 [file cancers-13-00975-s001.pdf]

Supplementary Materials: Identification of BXDC2 as a Key Downstream Effector of the Androgen Receptor in Modulating Cisplatin Sensitivity in Bladder Cancer

Guiyang Jiang, Yuki Teramoto, Takuro Goto, Taichi Mizushima, Satoshi Inoue, Hiroki Ide, Yujiro Nagata, Eiji Kashiwagi, Alexander S. Baras, George J. Netto, Zhiming Yang and Hiroshi Miyamoto

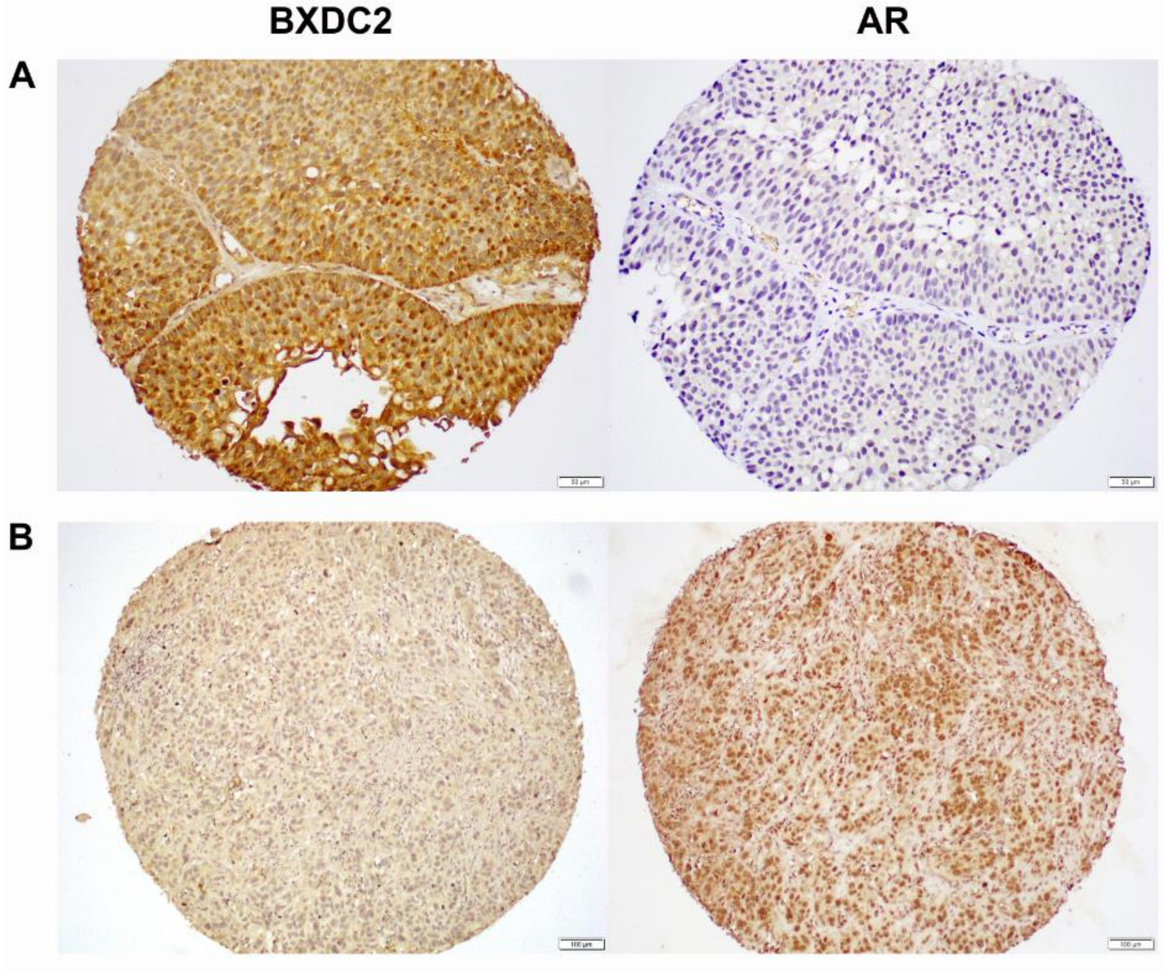

Figure S1. The expression of BXDC2 and AR in bladder cancer tissues. Strong immunoreactivity for BXDC2 vs. no nuclear immunoreactivity for AR (A, original magnification: 200x) and focal week immunoreactivity for BXDC2 vs. strong immunoreactivity for AR (B, original magnification: 100x) are seen in each identical core in the tissue microarray.

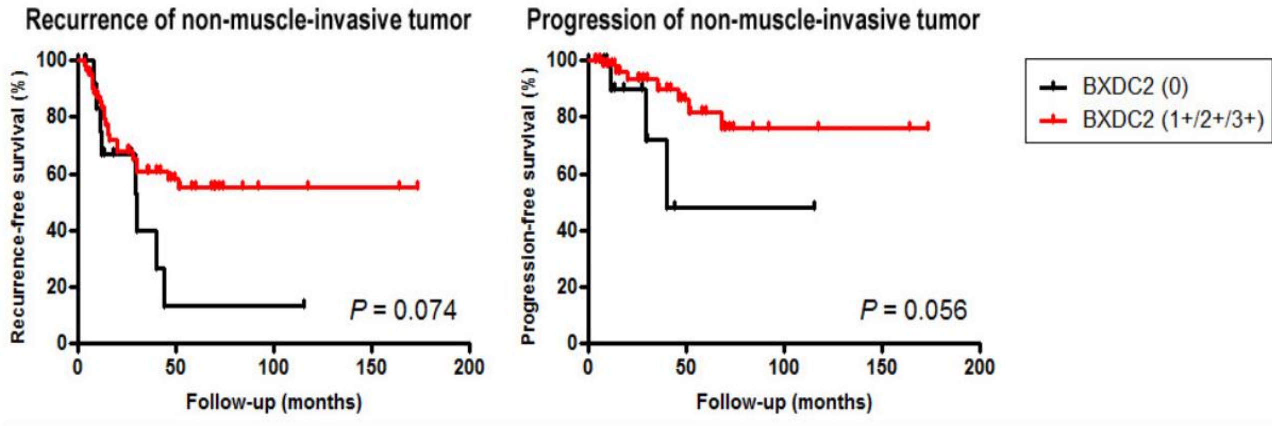

Figure S2. Kaplan–Meier analysis for recurrence-free survival or progression-free survival in patients with BXDC2-negative (n = 14) vs. BXDC2-positive (n = 64) non-muscle-invasive tumor.

Figure 1

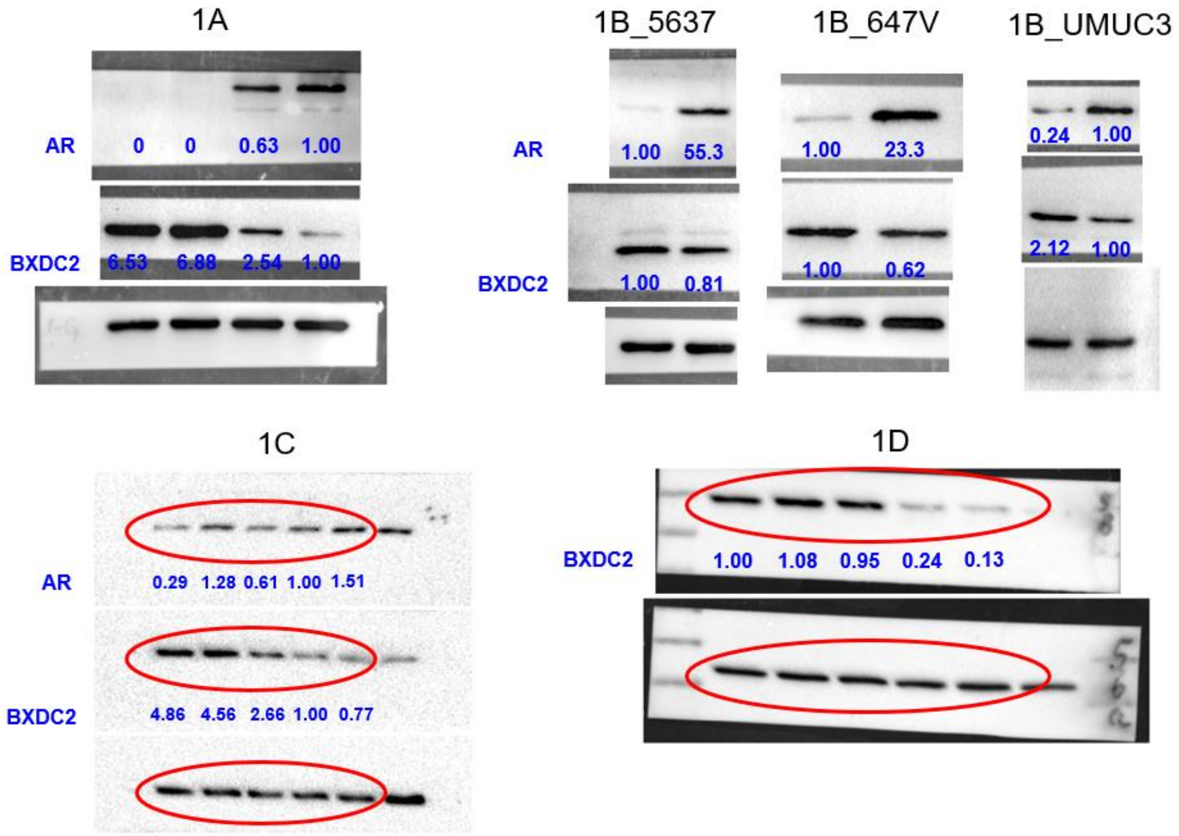

Figure 2

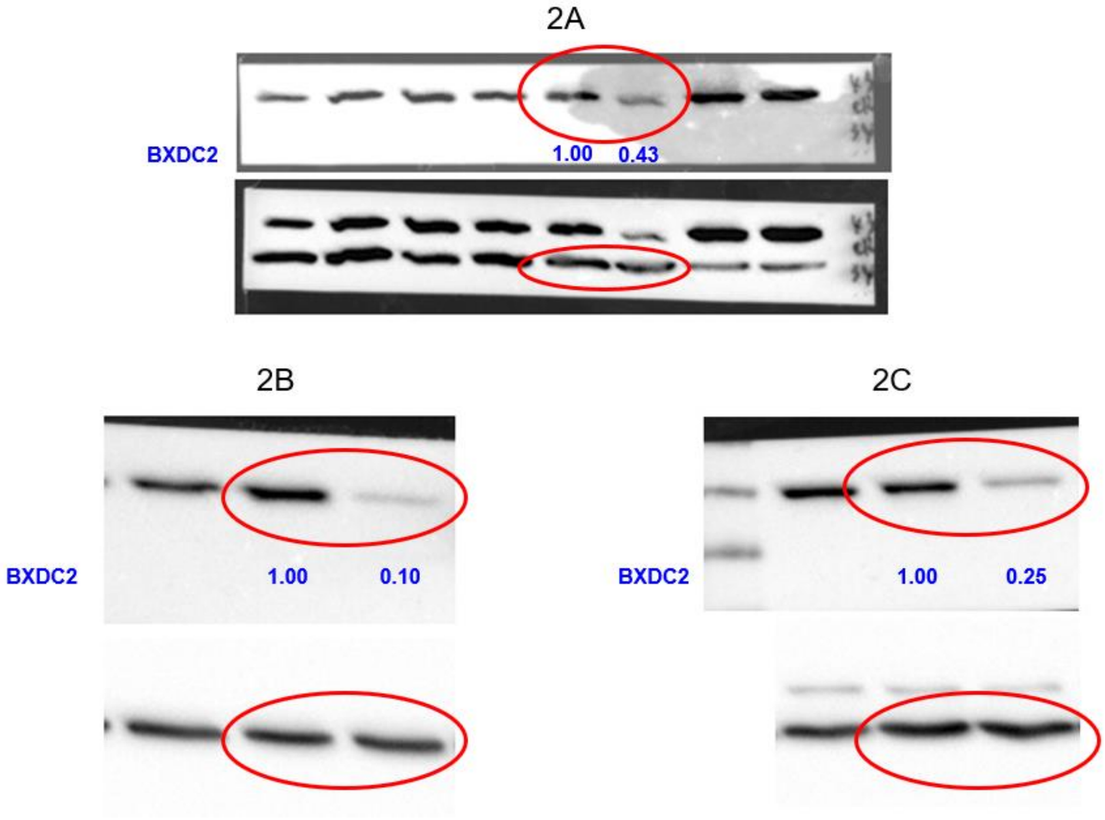

Figure 5

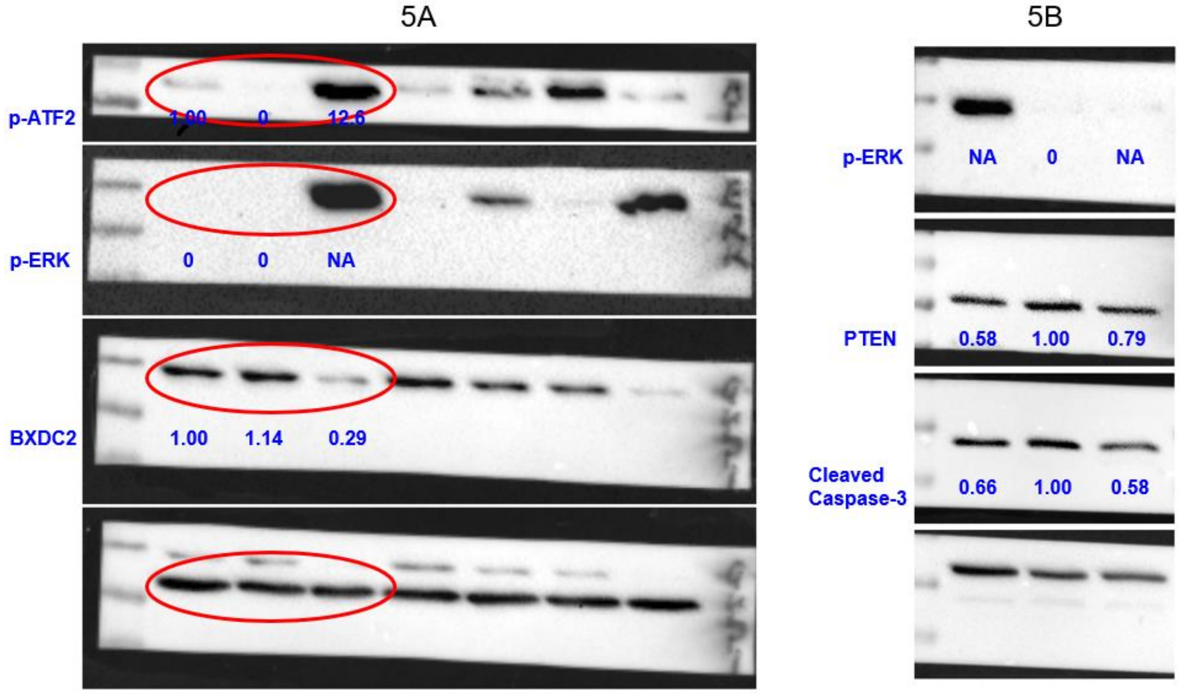

Figure S3. The whole Western blot images for Figures 1, 2, and 5. In all of these immunoblottings, the PVDF membrane was cut before incubation with primary antibodies, including those against AR, BXDC2, and GAPDH, as well as p-ATF2, p-ERK, PTEN, and cleaved caspase-3. Densitometry values

standardized by GAPDH (that are relative to those of control, mock treatment, or the other in each cell line or each immunoblot) are included below the lanes. NA, relative value not available.
